# Global epidemiology and mortality trends of necrotizing fasciitis: a systematic review and meta-analysis with a focus on monomicrobial Gram-negative infections

**DOI:** 10.1097/JS9.0000000000004744

**Published:** 2026-01-13

**Authors:** Yun-Shan Yeh, Pin-Jung Chen, Sheng-Chi Huang, Chin-Hao Chang, Hsin Yang, Tsae-Ni Lee, Chi-Tai Fang, Nai-Chen Cheng

**Affiliations:** a Division of Plastic Surgery, Department of Surgery, National Taiwan University Hospital and College of Medicine, National Taiwan University, Taipei, Taiwan; b School of Medicine, College of Medicine, National Taiwan University, Taipei, Taiwan; c Department of Medical Research, National Taiwan University Hospital, Taipei, Taiwan; d Institute of Epidemiology and Preventive Medicine, College of Public Health, National Taiwan University, Taipei, Taiwan; e Department of Urology, National Taiwan University Hospital, Taipei, Taiwan; f Department of Internal Medicine, National Taiwan University Hospital and College of Medicine, National Taiwan University, Taipei, Taiwan

## Abstract

**Background:**

Necrotizing fasciitis (NF) is a fulminant soft-tissue infection with high in-hospital mortality. Although NF is traditionally classified into types I–IV by microbiology, emerging data – particularly from East Asia – suggest that monomicrobial Gram-negative (mono-GNB) NF is epidemiologically and prognostically distinct. This systematic review and meta-analysis aimed to quantify global subtype distributions and in-hospital mortality, with a focus on mono-GNB NF.

**Methods:**

We searched PubMed/MEDLINE, Embase, Web of Science, and Cochrane Library from inception to 1 September 2023, for eligible studies reporting NF microbiology. Included cases were classified as polymicrobial, monomicrobial Gram-positive, mono-GNB, culture-negative, or other pathogen-related. The primary outcomes were the pooled proportion of mono-GNB NF cases and the associated in-hospital mortality. We used random-effects models to estimate pooled proportions and mortality rates, and we conducted subgroup analyses by time period (before 2000 vs. from 2000 onward) and the six World Health Organization (WHO) regions.

**Results:**

A total of 142 studies encompassing 6740 patients were included. Globally, the pooled proportion of mono-GNB NF increased from 12% before 2000 to 18% from 2000 onward (*P* = 0.03). Between-study heterogeneity was substantial (*I*^2^ > 95%). The proportion of mono-GNB cases was highest in the WHO regions of the Western Pacific, South-East Asia, and Africa. Among NF subtypes, mono-GNB NF exhibited the highest in-hospital mortality (28% before 2000 vs. 22% from 2000 onward; *P* = 0.70). Between-study heterogeneity was moderate (*I*^2^ = 41%).

**Conclusion:**

The rising proportion and persistently high in-hospital mortality of mono-GNB NF support its recognition as a distinct NF subtype and warrant reconsideration of the conventional NF classifications. In high-prevalence regions, empiric antimicrobial regimens should include prompt Gram-negative coverage to complement surgical debridement, while sustained epidemiological surveillance remains essential.

## Background

Necrotizing fasciitis (NF) is a fulminant, life-threatening soft-tissue infection characterized by rapid spread along fascial planes, causing extensive necrosis of the fascia and overlying subcutaneous tissue^[1–3]^. Although NF is relatively rare, with global incidence ranging from 0.86 to 32.64 cases per 100000 person-years^[4]^, its incidence has reportedly risen worldwide over recent decades^[2,4]^. Despite advances in care, mortality remains high (ranging from 16 to 34%). Adverse outcomes are associated with advanced age, comorbidities, immunocompromised status, clinical presentation, and disease severity^[2,5]^. Definitive management depends on prompt, aggressive surgical debridement combined with broad-spectrum antibiotic therapy^[2,4,6]^.

NF is conventionally classified into four microbiological types: Type I: polymicrobial (mixed aerobic–anaerobic, often in older or immunocompromised hosts); Type II: monomicrobial Gram-positive (mono-GPB), classically due to group A *Streptococcus* and occasionally *Staphylococcus aureus*; Type III: marine-associated Gram-negative pathogens (e.g., *Vibrio, Aeromonas*); and Type IV: fungal infections in severely immunocompromised hosts^[1,7,8]^. However, this classification does not adequately capture the growing burden of monomicrobial Gram-negative (mono-GNB) NF, including *Escherichia coli* and *Klebsiella pneumoniae*^[5,9–11]^.

Historically, a two-type framework – Type I (polymicrobial) and Type II (mono-GPB) – remains predominant in practice, whereas Types III and IV are less universally accepted^[1,3,7]^. This two-type framework lacks an explicit category for mono-GNB infections, creating a clinically relevant classification gap. However, recent reports from East Asia have revealed an increasing proportion of mono-GNB NF, with higher mortality rates compared to Types I and II^[5,9,10,12]^. Consequently, some experts have proposed revising the classification to formally recognize mono-GNB NF as a distinct subtype, given its unique epidemiological and prognostic profile^[5,9,11]^.

Although the emergence of mono-GNB NF has gained recognition in East Asia, its global relevance remains uncertain. Most available evidence comes from single-center series with limited geographic coverage, limiting generalizability.

To bridge this critical gap, we conducted a systematic review and meta-analysis to quantify the global distribution and in-hospital mortality of polymicrobial, mono-GPB, and mono-GNB NF across the six World Health Organization (WHO)-defined regions, and to compare periods before 2000 and from 2000 onward. We evaluated whether restructuring the Type III classification to prioritize mono-GNB NF over marine pathogens, while maintaining the latter as a subcategory, would improve clinical utility, facilitate accurate epidemiological surveillance, and better inform empiric antimicrobial management strategies globally.

## Materials and methods

We conducted this systematic review and meta-analysis in accordance with Preferred Reporting Items for Systematic reviews and Meta-Analyses (PRISMA 2020)^[13]^. The protocol was prospectively registered with PROSPERO. Methodological conduct was appraised using the A MeaSurement Tool to Assess systematic Reviews (AMSTAR 2)^[14]^. The study adhered to the Transparency In The reporting of Artificial INtelligence (TITAN) Guidelines 2025 to ensure transparent and responsible use of artificial intelligence^[15]^.

### Literature search strategy

We searched PubMed/MEDLINE (via PubMed), Embase (Elsevier), Web of Science, Cochrane Library, and Cochrane Central Register of Controlled Trials (CENTRAL) from inception to 1 September 2023, using the following keywords: “necrotizing fasciitis,” “necrotising fasciitis,” “necrotizing soft tissue infection,” and “necrotizing skin and soft tissue infection” without language restriction. Records were exported to EndNote 21 (Clarivate) for de-duplication (automated algorithm plus manual verification) before screening against prespecified eligibility criteria.

### Study selection process and eligibility criteria

Two reviewers independently screened titles/abstracts and full texts against prespecified eligibility criteria and classified eligible studies; disagreements were resolved by a senior investigator. The study selection process is summarized in a PRISMA flow diagram. Exclusion criteria at title/abstract screening were:

(a) non-English publications, owing to the difficulty of verifying duplicate records across languages; (b) non-original articles (conference proceedings/abstracts, theses, textbooks, book chapters, reviews); (c) case reports or case series with <10 patients; (d) *in vitro* or animal studies; (e) studies without a confirmed diagnosis of NF or not related to NF; and (f) studies restricted to a single-pathogen subset that precluded estimating overall subtype distributions.

HIGHLIGHTS

- The global proportion of mono-GNB NF rose from 12% pre-2000 to 18% post-2000 (*P* = 0.03).
- Mono-GNB NF had the highest in-hospital mortality (28% pre-2000; 22% post-2000; *P* = 0.70).
- Burden was greatest in the Western Pacific, South-East Asia, and Africa.
- Findings support formal Type III recognition and updated empiric protocols with stronger surveillance.
- For surgeons: in high-burden settings, begin early Gram-negative empiric cover per local antibiograms without delaying debridement; obtain an intraoperative Gram stain to adjust antibiotics promptly.

Studies that passed the title/abstract screening were read in full text. Those who met the following criteria were included in the meta-analysis:

(a) human participants with surgically confirmed NF, as defined by the original study (where reported, confirmation required intraoperative fascial necrosis and/or histopathology), excluding cellulitis, suspected NF without operative confirmation, or non-fascial necrotizing infections; (b) culture-confirmed microbiological diagnosis permitting assignment to predefined NF subtypes (polymicrobial, mono-GPB, mono-GNB, culture-negative, or other pathogen-related) and enabling estimation of subtype proportions; (c) a clearly defined study period enabling epoch stratification (before 2000 vs. from 2000 on); (d) country/region reported and mappable to a WHO region; and (e) original human research (e.g., cohort, case series) with sample size ≥10.

For overlapping cohorts from the same institution and period, we retained the largest or most comprehensive dataset according to a prespecified hierarchy (longer recruitment duration, more comprehensive microbiological data, and completeness of outcomes reporting).

### Risk-of-bias and quality assessment

Two reviewers independently appraised study validity, with disagreements adjudicated by a senior investigator. To assess risk of bias, we applied Cochrane RoB 2 for randomized trials, the Newcastle–Ottawa Scale (NOS) for cohort, case–control, and cross-sectional studies, the NIH Quality Assessment Tool for Case Series Studies for case series, and QUADAS-2 for diagnostic accuracy studies^[16–18]^. The certainty of evidence was summarized using the GRADE approach for the primary outcomes, with overall ratings of high, moderate, low, or very low reported at the outcome level^[17]^ (details in Supplemental Digital Content Table S2, available at: http://links.lww.com/JS9/G613).

We prespecified interpretation thresholds for NOS: 0–3 = high risk, 4–6 = moderate risk, and 7–9 = low risk. Individual domain judgments and overall ratings are provided in Supplemental Digital Content Table S2, available at: http://links.lww.com/JS9/G613. Risk-of-bias assessments informed interpretation of the pooled estimates; however, no studies were excluded based solely on these assessments.

### Data extraction

Two reviewers independently extracted data, including: first author; study initiation year and study period; country (mapped to WHO region); study design; sample size; mean age; sex distribution; NF microbiological subtype distribution; detailed organism profiles; and in-hospital mortality. For microbiological analyses, priority was given to deep tissue or intraoperative culture results when available; blood culture results were accepted when tissue cultures were not reported. Primary outcomes were the pooled proportion of mono-GNB among NF cases and in-hospital mortality; secondary analyses summarized regional and temporal patterns.

### Statistical analysis and classification

Descriptive analyses were performed in SPSS v25.0 (IBM, Armonk, New York). Data were stratified by the six WHO-defined regions: the Americas, the Western Pacific, Europe, the Eastern Mediterranean, South-East Asia, and Africa. NF subtypes were classified as polymicrobial, mono-GPB, mono-GNB, culture-negative, and other pathogen-related. Studies were further stratified by study initiation year (before 2000 vs. from 2000 on) to assess temporal trends in subtype distribution and in-hospital mortality. Group comparisons were performed on study-level aggregated data using χ^2^ tests (or Fisher’s exact tests when expected counts were <5) and *t*-tests where appropriate; significance was set at two-sided *α* = 0.05.

Regional subtype proportions and, where available, in-hospital mortality were calculated within each study and summarized by region/period. When only percentages were reported, absolute counts were back-calculated from study denominators if possible. Multicountry studies spanning a single WHO region were assigned to that region; studies spanning multiple WHO regions were excluded from the analyses.

### Meta-analysis

Random-effects meta-analyses were conducted in R (v4.4.2; R Foundation for Statistical Computing, Vienna, Austria) using the metafor package (v4.8-0) to pool subtype proportions and in-hospital mortality. Between-study heterogeneity was assessed using *I*^2^ and *τ*^2^ statistics. For studies with 0% or 100% event rates, a continuity correction of 0.5 was applied to stabilize variance. Leave-one-out sensitivity analyses were performed to assess the robustness and stability of all pooled effect estimates^[19]^. Subgroup differences across the WHO regions were assessed using Cochrane’s *Q* test for subgroup differences, calculated as the difference between overall heterogeneity and the sum of within-region heterogeneity, with statistical significance set at two-sided *α* = 0.05. 95% prediction intervals were reported for random-effects models where relevant.

Potential publication or small-study bias was examined with the Doi plot and Luis Furuya–Kanamori (LFK) index (not assessed when *k* < 10 studies; |LFK| ≤ 1 indicating no asymmetry, 1–2 minor asymmetry, and >2 major asymmetry)^[20]^. For clinical interpretability, confidence intervals that extended beyond logical boundaries (<0 or >1) were truncated to the nearest boundary (set to 0 or 1, respectively), without altering model estimates.

## Results

### Characteristics of included studies

**Figure 1.**
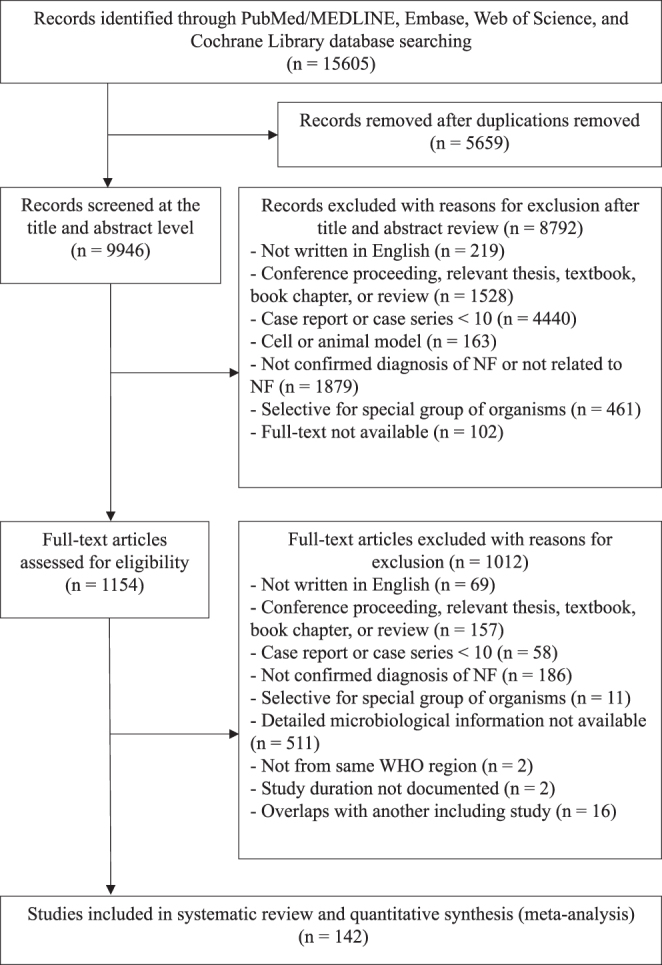
PRISMA 2020 flowchart.

Study selection is summarized in Figure 1. The database search identified 15605 records. After de-duplication in EndNote 21, titles and abstracts were screened for eligibility. After applying prespecified criteria, 1154 full-text articles were assessed; 142 studies (Supplemental Digital Content Table S1, available at: http://links.lww.com/JS9/G613) met inclusion criteria and were included in the systematic review, encompassing 6740 patients. After further exclusion of cases lacking sufficient microbiological data, 6245 patients were included in the meta-analysis.

Included studies were published between 1964 and 2023; demographic characteristics are summarized in Table 1. Overall, 4421 patients (67.5%) were male, and the mean age was 50.7 years. Of the included studies, 8 (5.6%) exclusively enrolled pediatric populations; 17 (12.0%) examined head-and-neck NF, 11 (7.7%) focused on extremity NF, and 20 (14.1%) specifically studied Fournier’s gangrene.

**Table 1:** Demographic characteristics of 142 included studies.

Demographic characteristics	Frequency	%
Gender		
Male	4421	67.5
Female	2128	32.5
Geographic regions		
Americas	33	23.2
Western Pacific	36	25.4
Europe	53	37.3
Eastern Mediterranean	8	5.6
Africa	3	2.1
South-East Asian	9	6.3
Time the study began		
Before 2000	55	38.7
After 2000	87	61.3
Studies focus on specific subgroup		
Pediatric NF	8	5.6
Head and neck NF	17	12.0
Extremities NF	11	7.7
Fournier’s gangrene	20	14.1
Other	86	60.7

**Figure 2.**
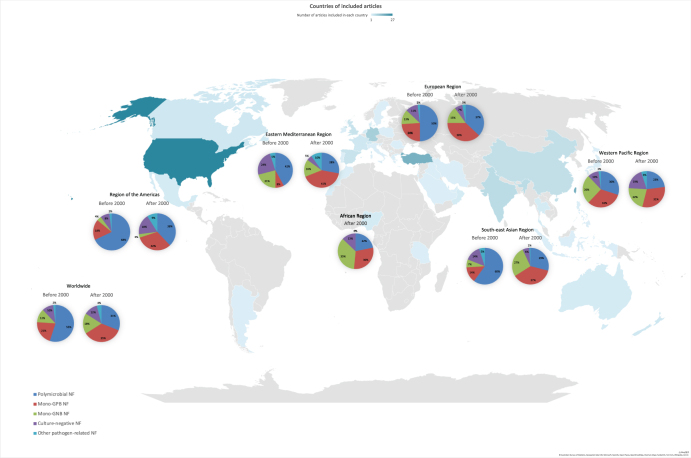
Global NF type proportions map.

**Figure 3.**
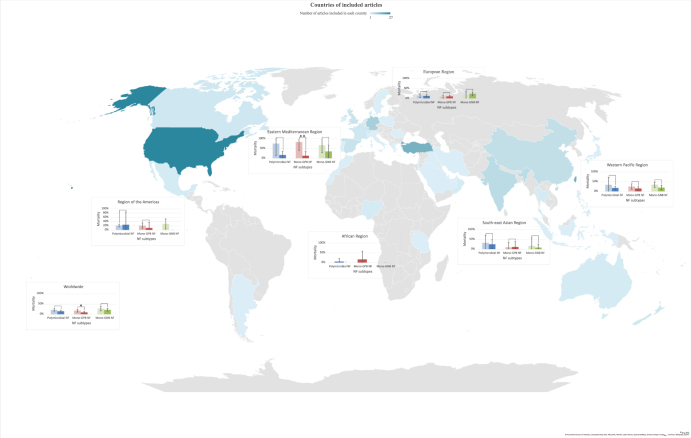
Global NF type mortality map.

Studies were categorized by the six WHO-defined regions. Specifically, 33 studies were from the Americas, 36 from the Western Pacific, 53 from Europe, 8 from the Eastern Mediterranean, 3 from Africa, and 9 from South-East Asia. Figure 2 depicts the geographic distribution of included studies by country alongside pooled regional proportions of NF subtypes. Using the same geographic layout, Figure 3 presents pooled in-hospital mortality for each NF subtype across WHO regions.

Geographic distribution of included studies by country, overlaid with the pooled proportion of NF subtypes across WHO-defined regions.

Geographic distribution of included studies by country, overlaid with the pooled in-hospital mortality rates of NF types I, II, and III across WHO-defined regions. Error bars represent 95% confidence intervals. Asterisks indicate statistically significant regional differences (*p < 0.05, **p < 0.01).

### Temporal comparison of NF subtype proportions across geographic regions

**Figure 4.**
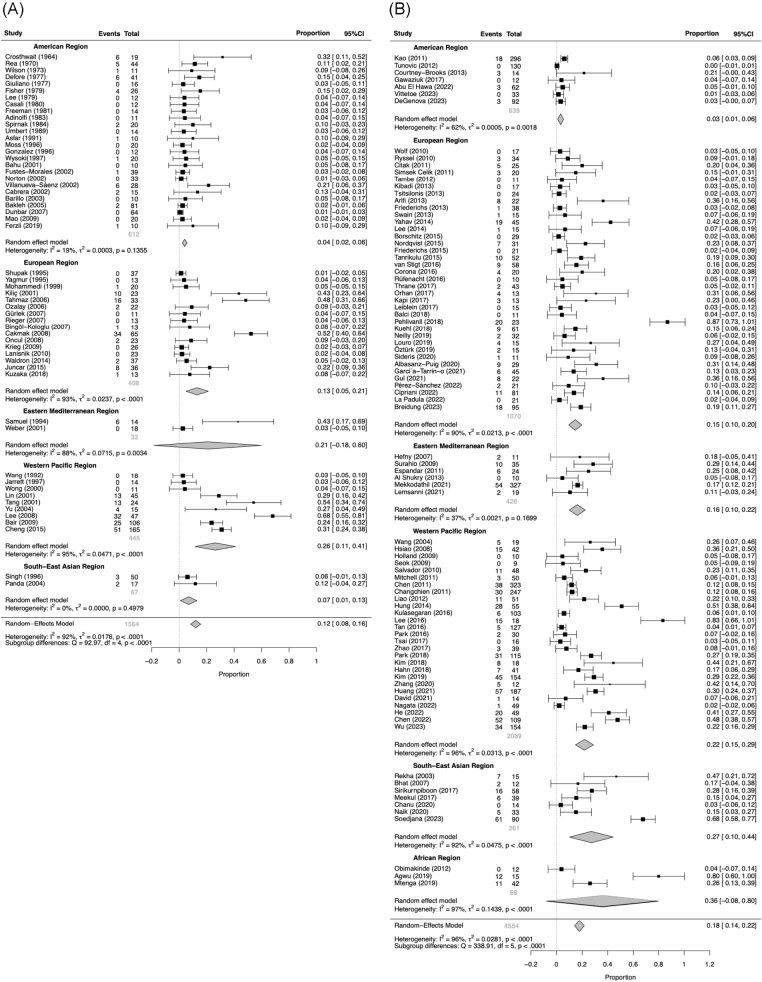
Forest plots of mono-GNB NF proportion.

**Figure 5.**
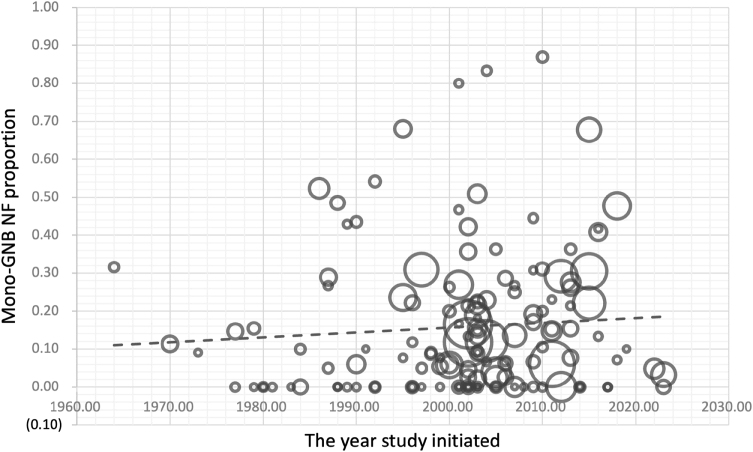
Bubble plot of mono-GNB NF proportions.

Globally, before 2000, polymicrobial NF predominated, with a pooled proportion of 0.55 (95% CI, 0.46–0.64; *I*^2^ = 98%) under a random-effects model. Mono-GPB and mono-GNB NF were less frequent, with pooled proportions 0.21 (95% CI, 0.16–0.26; *I*^2^ = 91%) and 0.12 (95% CI, 0.08–0.16; *I*^2^ = 92%), respectively. Culture-negative NF had a pooled proportion of 0.10 (95% CI, 0.06–0.14; *I*^2^ = 94%). From 2000 onward, subtype distributions shifted. The proportion of polymicrobial NF decreased to 0.31 (95% CI, 0.26–0.36; *I*^2^ = 98%), while mono-GPB NF increased to 0.35 (95% CI, 0.31–0.40; *I*^2^ = 95%). Notably, the proportion of mono-GNB NF rose significantly to 0.18 (95% CI, 0.14–0.22; *I*^2^ = 96%; *P* = 0.03), as illustrated in Supplemental Digital Content Figure S1, available at: http://links.lww.com/JS9/G612; the detailed regional distribution of this subtype is illustrated in Figure 4. Culture-negative NF increased slightly to 0.12 (95% CI, 0.09–0.16; *I*^2^ = 98%). See Supplemental Digital Content Figures S2–S4, available at: http://links.lww.com/JS9/G612 for additional subtype-specific results. Comprehensive summary statistics for all NF subtype proportions are provided in Supplemental Digital Content Table S3, available at: http://links.lww.com/JS9/G613. Pooled estimates exhibited substantial between-study heterogeneity (*I*^2^ = 91–98%). Leave-one-out sensitivity analyses showed no meaningful change in this pooled estimate (Supplemental Digital Content Figures S5–S8, available at: http://links.lww.com/JS9/G612). Findings are summarized in Table 2 and visualized in Figure 5 as a study-level bubble plot depicting mono-GNB NF proportions over time, with bubble size proportional to sample size. See Supplemental Digital Content Figures S9–S11, available at: http://links.lww.com/JS9/G612 for detailed analyses of other NF subtypes.

Forest plots from the random-effects meta-analysis showing the pooled proportion of mono-GNB NF before and after 2000. (A) Studies conducted prior to 2000. (B) Studies conducted in or after 2000.

Study-level bubble plot illustrating the temporal distribution of mono-GNB NF proportions. Each bubble represents an individual study, with its position reflecting the year of study initiated and reported mono-GNB NF proportion, and its size corresponding to the study’s sample size.

**Table 2:** Summary statistics of NF subtype proportions worldwide.

Dependent variable	Study number	Case number	Pooled proportion estimates [95% CI]	*I*^2^	*τ*^2^	*P*
Before 2000 AD	**55**					
Polymicrobial NF		692	0.55 [0.46, 0.64]	98%	0.1071	<0.0001
Mono-GPB NF		362	0.21 [0.16, 0.26]	91%	0.0289	<0.0001
Mono-GNB NF		264	0.12 [0.08, 0.16]	92%	0.0176	<0.0001
Culture-negative NF		200	0.10 [0.06, 0.14]	94%	0.0191	<0.0001
After 2000 AD	**87**					
Polymicrobial NF		1341	0.31 [0.26, 0.36]	98%	0.0503	<0.0001
Mono-GPB NF		1577	0.35 [0.31, 0.40]	95%	0.0470	<0.0001
Mono-GNB NF		826	0.18 [0.14, 0.22]	96%	0.0281	<0.0001
Culture-negative NF		829	0.12 [0.09, 0.16]	98%	0.0246	<0.0001

Region of the Americas – Before 2000, polymicrobial NF predominated (0.68; 95% CI, 0.57–0.79; *I*^2^ = 96%). Mono-GPB NF and mono-GNB NF accounted for 0.18 (95% CI, 0.11–0.24; *I*^2^ = 90%) and 0.04 (95% CI, 0.02–0.06; *I*^2^ = 19%), respectively, while culture-negative NF was 0.08 (95% CI, 0.03–0.14; *I*^2^ = 93%). From 2000 onward, the proportion of polymicrobial NF declined to 0.38 (95% CI, 0.18–0.58; *I*^2^ = 97%), whereas mono-GPB increased to 0.32 (95% CI, 0.22–0.43; *I*^2^ = 85%), mono-GNB NF remained uncommon (0.03; 95% CI, 0.01–0.06; *I*^2^ = 62%), while culture-negative NF increased to 0.18 (95% CI, 0.04–0.32; *I*^2^ = 98%).

European Region – Before 2000, polymicrobial NF was the predominant subtype (0.50; 95% CI, 0.33–0.67; *I*^2^ = 98%). Mono-GPB and mono-GNB NF were less common, accounting for 0.24 (95% CI, 0.13–0.35; *I*^2^ = 94%) and 0.13 (95% CI, 0.05–0.21; *I*^2^ = 93%), respectively, while culture-negative NF was 0.11 (95% CI, 0.02–0.20; *I*^2^ = 96%). From 2000 onward, the proportion of polymicrobial NF declined to 0.37 (95% CI, 0.29–0.44; *I*^2^ = 95%), mono-GPB NF increased to 0.38 (95% CI, 0.29–0.47; *I*^2^ = 96%), becoming the most common subtype. Mono-GNB NF also rose slightly to 0.15 (95% CI, 0.10–0.20; *I*^2^ = 90%), while culture-negative NF decreased to 0.07 (95% CI, 0.03–0.11; *I*^2^ = 95%).

Eastern Mediterranean Region – Before 2000, evidence was limited (two studies), yielding imprecise estimates with wide confidence intervals. Polymicrobial NF was the predominant subtype (0.42; 95% CI, 0–1.00; *I*^2^ = 97%). Pooled proportions for mono-GPB and mono-GNB were 0.08 (95% CI, 0–0.17; *I*^2^ = 0%) and 0.21 (95% CI, 0–0.60; *I*^2^ = 88%), respectively, while culture-negative was 0.24 (95% CI, 0.06–0.42; *I*^2^ = 34%), precluding firm inference. From 2000 onward, the distribution became more clearly defined across eight studies: mono-GPB emerged as the most common (0.41; 95% CI, 0.23–0.60; *I*^2^ = 90%), followed by polymicrobial (0.28; 95% CI, 0.15–0.41; *I*^2^ = 82%), mono-GNB (0.16; 95% CI, 0.10–0.22; *I*^2^ = 37%), and culture-negative (0.05; 95% CI, 0.01–0.08; *I*^2^ = 41%).

Western Pacific Region – Before 2000, mono-GPB NF was most common (0.32; 95% CI, 0.23–0.41; *I*^2^ = 72%), followed by polymicrobial NF (0.30; 95% CI, 0.08–0.52; *I*^2^ = 99%) and mono-GNB NF (0.26; 95% CI, 0.11–0.41; *I*^2^ = 95%); culture-negative NF was 0.10 (95% CI, 0.05–0.15; *I*^2^ = 71%). From 2000 onward, polymicrobial NF declined to 0.23 (95% CI, 0.15–0.30; *I*^2^ = 98%). Mono-GPB NF remained stable at 0.31 (95% CI, 0.25–0.38; *I*^2^ = 94%), mono-GNB decreased slightly to 0.22 (95% CI, 0.15–0.29; *I*^2^ = 96%), and culture-negative increased to 0.19 (95% CI, 0.11–0.27; *I*^2^ = 99%).

South-East Asian Region – A marked temporal shift in NF subtype distribution was observed. Before 2000, polymicrobial NF accounted for most cases (0.60; 95% CI, 0.14–1.00; *I*^2^ = 92%). Mono-GPB and mono-GNB were less common, at 0.14 (95% CI, 0.04–0.25; *I*^2^ = 32%) and 0.07 (95% CI, 0–0.13; *I*^2^ = 0%), respectively, while culture-negative was 0.14 (95% CI, 0–0.40; *I*^2^ = 83%). From 2000 onward, mono-GNB increased to 0.27 (95% CI, 0.10–0.44; *I*^2^ = 92%), mono-GPB was 0.37 (95% CI, 0.27–0.46; *I*^2^ = 95%), while polymicrobial declined to 0.29 (95% CI, 0.04–0.53; *I*^2^ = 99%). Culture-negative remained uncommon (0.06; 95% CI, 0.02–0.11; *I*^2^ = 64%).

African Region – No eligible data were available before 2000. From 2000 onward, mono-GNB was the most prevalent subtype (0.36; 95% CI, 0.08–0.80; *I*^2^ = 97%), followed by mono-GPB (0.30; 95% CI, 0.08–0.52; *I*^2^ = 75%); polymicrobial and culture-negative were less frequent, at 0.22 (95% CI, 0–0.62; *I*^2^ = 92%) and 0.13 (95% CI, 0–0.27; *I*^2^ = 69%), respectively. Estimates were imprecise because of limited studies and wide confidence intervals.

### Regional differences in mono-GNB NF proportion

Before 2000 – Significant between-region variation was observed (*Q* = 92.97, df = 4, *P* < 0.0001). Pooled proportions were higher in the Western Pacific (0.26; 95% CI, 0.11–0.41) and the Eastern Mediterranean (0.21; imprecise), lower in the Americas (0.04; 95% CI, 0.02–0.06) Europe (0.13; 95% CI, 0.05–0.21), and South-East Asia (0.07; 95% CI, 0.01–0.13). These differences should be interpreted cautiously because pre-2000 data were sparse in several regions and confidence intervals were wide. (Africa had no eligible pre-2000 data and was not included in this test.)

From 2000 onward – Regional differences persisted significantly (*Q* = 338.91, df = 5, *P* < 0.0001). Mono-GNB proportions were highest in Africa (0.36; wide CI) and South-East Asia (0.27), intermediate in the Western Pacific (0.22) and the Eastern Mediterranean (0.16), and lowest in the Americas (0.03) and Europe (0.15). Despite statistical significance, confidence intervals overlapped across several regions, and within-region heterogeneity remained substantial, particularly in Africa; therefore, these findings reflect average tendencies rather than discrete, nonoverlapping regional profiles. Overall, the pattern is consistent with a greater mono-GNB burden in parts of South-East Asia, the Western Pacific, and Africa relative to the Americas and Europe.

### Temporal comparison of in-hospital mortality across geographic regions

**Figure 6.**
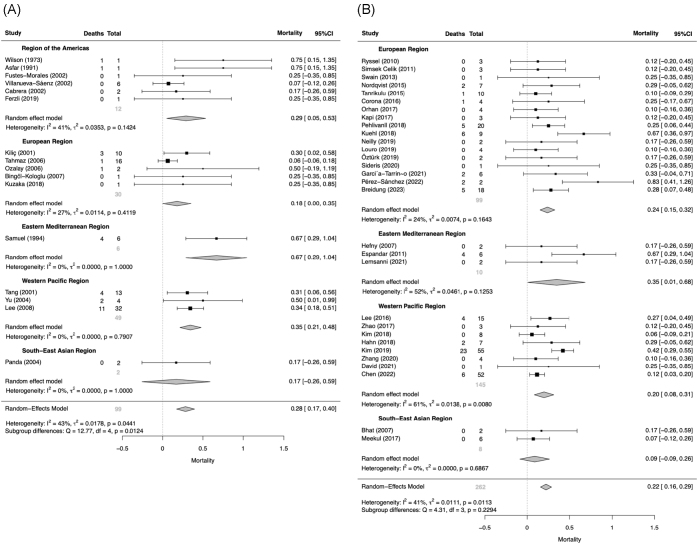
Forest plots of mono-GNB NF mortality.

**Figure 7.**
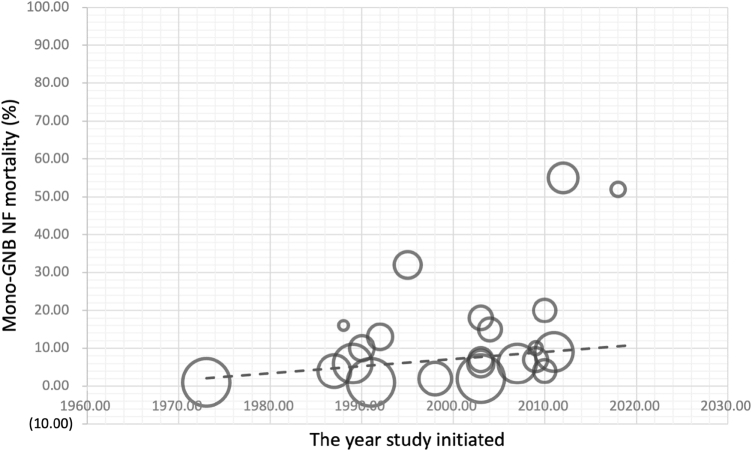
Bubble plot of mono-GNB NF mortality.

Globally, mono-GNB NF had the highest in-hospital mortality in both epochs. Before 2000, pooled mortality was 0.28 (95% CI, 0.17–0.40; *I*^2^ = 43%), exceeding polymicrobial 0.22 (95% CI, 0.15–0.29; *I*^2^ = 66%) and mono-GPB 0.18 (95% CI, 0.11–0.24; *I*^2^ = 15%). From 2000 onward, mortality decreased modestly across subtypes; mono-GNB remained highest at 0.22 (95% CI, 0.16–0.29; *I*^2^ = 41%), compared with polymicrobial 0.15 (95% CI, 0.11–0.20; *I*^2^ = 38%) and mono-GPB 0.10 (95% CI, 0.07–0.14; *I*^2^ = 33%). Figure 6 presents mono-GNB mortality; results for other subtypes are shown in Supplemental Digital Content Figures S12 and S13, available at: http://links.lww.com/JS9/G612. Heterogeneity was generally moderate across subtypes in both epochs. Findings are summarized in Table 3. Summary statistics for in-hospital mortality across all NF subtypes and regions are detailed in Supplemental Digital Content Table S4, available at: http://links.lww.com/JS9/G613. Leave-one-out sensitivity analyses showed no meaningful change in this pooled estimate (Supplemental Digital Content Figures S14–S16, available at: http://links.lww.com/JS9/G612). Figure 7 visualizes the persistent lethality of mono-GNB NF in a study-level bubble plot of in-hospital mortality over time, with bubble size proportional to sample size; detailed analyses for other subtypes are provided in Supplemental Digital Content Figures S17 and S18, available at: http://links.lww.com/JS9/G612.

Forest plots from the random-effects meta-analysis showing the pooled in-hospital mortality of mono-GNB NF before and after 2000. (A) Studies conducted prior to 2000. (B) Studies conducted in or after 2000.

Study-level bubble plot illustrating the temporal distribution of mono-GNB NF in-hospital mortality. Each bubble represents an individual study, with its position reflecting the year of study initiated and reported mono-GNB NF in-hospital mortality rate, and its size corresponding to the study’s sample size.

**Table 3:** Summary statistics of NF subtype intra-hospital mortality worldwide.

Dependent variable	Study number	Case number	Pooled mortality estimates [95%CI]	*I*^2^	*τ*^2^	*P*
Before 2000 AD						
Polymicrobial NF	29	323	0.22 [0.15, 0.29]	66%	0.0194	<0.0001
Mono-GPB NF	22	117	0.18 [0.11, 0.24]	15%	0.0036	0.3568
Mono-GNB NF	16	99	0.28 [0.17, 0.40]	43%	0.0178	0.0441
After 2000 AD						
Polymicrobial NF	39	362	0.15 [0.11, 0.20]	38%	0.0057	0.0283
Mono-GPB NF	42	437	0.10 [0.07, 0.14]	33%	0.0027	0.0428
Mono-GNB NF	30	262	0.22 [0.16, 0.29]	41%	0.0111	0.0113

Americas – Before 2000, in-hospital mortality was comparable across subtypes: polymicrobial 0.22 (95% CI, 0.15–0.28; *I*^2^ = 24%), mono-GPB 0.21 (95% CI, 0.08–0.34; *I*^2^ = 0%), and mono-GNB 0.29 (95% CI, 0.05–0.53; *I*^2^ = 41%). From 2000 onward, only isolated case data were available (*n* ≤ 1 per subtype), precluding pooled estimates and formal between-subtype comparisons. Within the pre-2000 epoch, mono-GNB showed the numerically highest mortality, though precision was limited by wide confidence intervals.

European Region – Before 2000, in-hospital mortality was similar across subtypes: mono-GNB 0.18 (95% CI, 0–0.35; *I*^2^ = 27%), polymicrobial 0.14 (95% CI, 0.04–0.23; *I*^2^ = 53%), and mono-GPB 0.09 (95% CI, 0.01–0.17; *I*^2^ = 0%). From 2000 onward, mono-GNB mortality increased to 0.24 (95% CI, 0.15–0.32; *I*^2^ = 24%), remaining the highest among subtypes, whereas polymicrobial and mono-GPB were lower at 0.13 (95% CI, 0.08–0.32; *I*^2^ = 25%) and 0.11 (95% CI, 0.07–0.15; *I*^2^ = 15%), respectively.

Eastern Mediterranean Region – Before 2000, estimates were imprecise because of small sample sizes; in-hospital mortality was mono-GNB 0.67 (95% CI, 0.29–1.00; *I*^2^ = 0%), polymicrobial 0.75 (95% CI, 0.15–1.00; *I*^2^ = 0%), and mono-GPB 0.83 (95% CI, 0.41–1.00; *I*^2^ = 0%). From 2000 onward, mono-GNB had the highest mortality at 0.35 (95% CI, 0.01–0.68; *I*^2^ = 52%), whereas polymicrobial and mono-GPB were lower at 0.17 (95% CI, 0.01–0.34; *I*^2^ = 0%) and 0.13 (95% CI, 0–0.34; *I*^2^ = 36%), respectively. Given sparse data and wide confidence intervals, these estimates should be interpreted with caution.

Western Pacific Region – Mono-GNB NF maintained the highest in-hospital mortality across epochs. Before 2000, mortality was 0.35 (95% CI, 0.21–0.48; *I*^2^ = 0%), exceeding mono-GPB 0.25 (95% CI, 0.12–0.39; *I*^2^ = 0%) and approximating polymicrobial 0.34 (95% CI, 0–0.72; *I*^2^ = 89%); the wide interval and high heterogeneity for polymicrobial warrant caution. From 2000 onward, mono-GNB remained elevated at 0.20 (95% CI, 0.08–0.31; *I*^2^ = 61%), compared with 0.18 for polymicrobial (95% CI, 0.08–0.27; *I*^2^ = 59%) and 0.15 for mono-GPB (95% CI, 0.08–0.27; *I*^2^ = 59%). Heterogeneity was moderate across subtypes.

South-East Asian Region – Before 2000, mortality estimates were derived from single-study reports with wide uncertainty: polymicrobial 0.33 (95% CI, 0–0.71), mono-GNB 0.17 (95% CI, 0–0.59), and mono-GPB 0.10 (95% CI, 0–0.36). From 2000 onward, mono-GNB mortality was 0.09 (95% CI, 0–0.26; *I*^2^ = 0%); polymicrobial remained relatively high at 0.26 (95% CI, 0.04–0.49; *I*^2^ = 19%), and mono-GPB was 0.12 (95% CI, 0–0.26; *I*^2^ = 63%). Interpretation remains cautious due to limited data.

African Region – No eligible data were available before 2000. From 2000 onward, estimates were based on two studies: polymicrobial mortality 0.06 (95% CI, 0–0.21; *I*^2^ = 0%) and mono-GPB 0.17 (95% CI, 0–0.59; *I*^2^ = 0%). No mortality data for mono-GNB were available, precluding pooled estimates and between-subtype comparisons.

### Regional differences in mono-GNB NF in-hospital mortality

Before 2000 – Subgroup analyses indicated significant regional variation in mono-GNB mortality (*Q* = 12.77, df = 4, *P* = 0.0124). Mortality was higher in the Eastern Mediterranean (0.67; wide CI) and Western Pacific (0.35), intermediate in the Americas (0.29), and lower in Europe (0.18) and South-East Asia (0.17). Interpretation should remain cautious due to sparse data; Africa had no data.

From 2000 onward – No significant between-region differences were observed (*Q* = 4.31, df = 3, *P* = 0.229). Mortality was numerically higher in the Eastern Mediterranean (0.35) and Europe (0.24) than in the Western Pacific (0.20) and South-East Asia (0.09), but confidence intervals overlapped and within-region heterogeneity persisted; thus, these results reflect average tendencies rather than discrete, nonoverlapping regional profiles. Data from the Americas and Africa were insufficient for inclusion in this comparison.

Overall, mono-GNB NF demonstrated persistently high in-hospital mortality with no significant temporal improvement (28% before 2000 vs. 22% from 2000 onward; *P* = 0.70). Between-study heterogeneity was moderate (*I*^2^ = 41%), as illustrated in Supplemental Digital Content Figure S19, available at: http://links.lww.com/JS9/G612.

### Risk of bias and certainty of evidence

Most included studies were retrospective, single-center case series or observational studies, resulting in variable overall quality. Common methodological limitations included potential selection bias, non-standardized definitions of NF subtypes, incomplete microbiological sampling methods, inadequate control for confounding factors, and missing data. Consequently, between-study heterogeneity was substantial, and small-study effects could not be excluded. Doi plots revealed minor asymmetry indicative of possible publication bias or small-study effects for analyses conducted both before and after 2000, as detailed in Table 4. Individual subtype plots illustrating these potential biases are presented in Supplemental Digital Content Figures S20–S27, available at: http://links.lww.com/JS9/G612.

**Table 4:** Summary statistics of publication bias.

Dependent variable	Doi plot	LFK index
Before 2000 AD		
Polymicrobial NF	Minor asymmetry	1.363
Mono-GPB NF	Minor asymmetry	1.354
Mono-GNB NF	Minor asymmetry	1.131
Culture-negative NF	No asymmetry	0.767
After 2000 AD		
Polymicrobial NF	Minor asymmetry	1.278
Mono-GPB NF	No asymmetry	0.990
Mono-GNB NF	Minor asymmetry	1.144
Culture-negative NF	No asymmetry	0.848

Applying the GRADE approach, the overall certainty of evidence for the primary outcomes (subtype proportions and in-hospital mortality rates) was generally low to very low. Reasons for downgrading included serious risk of bias (predominantly observational study designs with unadjusted estimates), high inconsistency (substantial heterogeneity indicated by high *I*^2^ statistics and directionally inconsistent effects), indirectness (single-center studies limiting generalizability and variable regional patient populations), and imprecision (wide confidence intervals and sparse data in certain subgroups). Where subgroup analyses included fewer than 10 studies (*k* < 10), evaluation of small-study effects was not performed due to limited interpretability. Comprehensive risk-of-bias judgments and detailed GRADE evidence profiles are provided in Supplemental Digital Content Table S2, available at: http://links.lww.com/JS9/G613.

## Discussion

In this meta-analysis of 142 studies, we delineate the global distribution and in-hospital mortality of NF subtypes across regions and time periods, providing a comprehensive regional and temporal comparison. Across WHO regions, the proportion of mono-GNB NF increased significantly from 2000 onward, rising globally from 12% before 2000 to 18% thereafter (*P* = 0.03). Despite modest reductions in absolute mortality over time (28% before 2000 vs. 22% after 2000; *P* = 0.70), mono-GNB NF consistently demonstrated the highest fatality among all subtypes. This epidemiologic shift was most pronounced in the Western Pacific, South-East Asia, and Africa, which signals a mounting global burden attributable to Gram-negative pathogens.

The persistently high in-hospital mortality of mono-GNB NF underscores its growing clinical significance. Compared with polymicrobial or mono-GPB NF, mono-GNB infections often present with subtle local signs and rapid systemic deterioration, leading to diagnostic delay and poor outcomes^[5,11,21,22]^. Pathogens such as *E. coli, P. aeruginosa*, and *K. pneumoniae* exhibit high virulence, potent endotoxin-mediated systemic effects, and frequent multidrug resistance, features that complicate early management^[23–27]^. Prompt surgical intervention, aggressive resuscitation, and early microbiology-guided therapy remain cornerstones of management. In endemic regions of mono-GNB NF, such as South-East Asia and the Western Pacific, empirical regimens should reflect local resistance patterns and may require broad-spectrum coverage with third-generation cephalosporins, β-lactam/β-lactamase inhibitor combinations, or carbapenems^[2,4,6,28,29]^.

The conventional classification fails to explicitly distinguish mono-GNB NF as a discrete entity. Although Type I (polymicrobial) and Type II (mono-GPB) categories remain clinically relevant, our findings aligned with data reported in recent studies and supported recognizing mono-GNB NF as a distinct subtype^[9–11]^. Prior proposals to designate mono-GNB NF as a revised Type III, with marine-associated *Vibrio* infections retained as a subcategory, merit consideration^[5,9]^. Our findings support this updated classification, which could improve early clinical recognition, optimize empiric antimicrobial selection, and strengthen epidemiological tracking.

Marked regional disparities in NF subtype distribution and mortality likely reflect differences in healthcare infrastructure, diagnostic turnaround, antimicrobial stewardship, and host comorbidities. In high-income regions, enhanced surveillance may explain the relative decline in polymicrobial NF, while in low- and middle-income settings, delays in presentation and limited culture capacity may obscure accurate classification^[2,28,30,31]^. Moreover, diabetes mellitus, liver cirrhosis, and immunosuppression are more commonly associated with mono-GNB NF and may explain its rising incidence, particularly in Asia^[3,32]^. Environmental factors, including increasing temperatures and water exposure, have also been linked to the spread of Gram-negative pathogens^[33]^.

Comparable Gram-negative predominance is emerging in other necrotizing and nosocomial infections, including Fournier’s gangrene, ventilator-associated pneumonia, and urosepsis – conditions where virulence and multidrug resistance contribute substantially to mortality^[34–37]^. Similarly, in nosocomial infections such as ventilator-associated pneumonia and urosepsis, Gram-negative organisms have also increased substantially and contribute disproportionately to mortality owing to their virulence and antibiotic resistance^[38,39]^. These parallels underscore a broader shift in pathogen ecology, reinforcing the need for vigilant surveillance and adapted management strategies.

Our findings have direct relevance for both clinical management and policy formulation. First, empiric antimicrobial regimens should be informed by regional epidemiology, with early Gram-negative coverage prioritized in areas where mono-GNB NF predominates. Second, heightened clinical vigilance, supported by rapid diagnostics such as Gram staining or molecular assays, may enable earlier intervention and improved outcomes. Third, standardizing NF classification and microbiologic reporting in both research and practice would enhance interstudy comparability and strengthen surveillance accuracy. Finally, the integration of NF surveillance into broader antimicrobial stewardship frameworks is essential, given the escalating antimicrobial resistance observed in Gram-negative NF pathogens. These data also underscore the urgency of developing novel therapeutic strategies and preventive measures targeting high-risk regions.

This meta-analysis is subject to several limitations. First, most included studies were retrospective, single-center series, introducing potential selection and reporting biases. Although we applied validated quality appraisal tools (e.g., NIH Case Series Tool, GRADE), the overall certainty of evidence – particularly in pre-2000 studies – remains limited. Second, beyond genuine clinical and epidemiological diversity across WHO regions, the substantial between-study heterogeneity (*I*^2^ often >90%) may also reflect variations in diagnostic criteria for NF, healthcare infrastructure and resources, and microbiological methodologies. Inconsistent reporting of pathogens further limited accuracy, as some studies did not distinguish polymicrobial from monomicrobial infections or Gram-positive from Gram-negative isolates. Culture-negative cases – often due to prior antibiotic exposure or suboptimal sampling – added further uncertainty. Third, data scarcity in regions such as Africa, South-East Asia, and the Eastern Mediterranean led to wide confidence intervals and reduced power for temporal and inter-subtype comparisons. Despite these constraints, our study represents the most comprehensive synthesis to date of NF subtype epidemiology and mortality, offering a robust foundation for future research, classification refinement, and targeted global surveillance efforts.

## Conclusions

This systematic review and meta-analysis delineates a global epidemiologic transition in NF, marked by a decline in polymicrobial cases and a rise in monomicrobial infections – most notably mono-GNB NF – in recent decades. The disproportionate increase in mono-GNB NF within South-East Asia, the Western Pacific, and Africa, coupled with its consistently highest in-hospital mortality, underscores its emerging significance as a distinct clinical subtype. These findings challenge the adequacy of traditional NF classifications and emphasize the need to integrate regional microbiological patterns into empiric treatment protocols. Prioritizing early recognition through enhanced clinician awareness, rapid diagnostic strategies, and targeted empiric coverage is imperative. For example, intraoperative Gram staining of wound discharge may provide an early indication of Gram-negative involvement, enabling timely adjustment of empiric antimicrobial therapy to complement surgical debridement and potentially improve overall outcomes. Establishing prospective, multicenter registries and standardized global surveillance will be critical to validate these observations, refine prognostic models, and guide the development of tailored preventive and therapeutic interventions.

## Data Availability

All data supporting the findings of this study were extracted from previously published articles included in the systematic review. No individual patient data were used, and no new datasets were generated. Data extracted for this meta-analysis are available from the corresponding author upon reasonable request.

## Research registration unique identifying number (UIN)

(1) Name of the registry: PROSPERO. (2) Unique identifying number or registration ID: CRD420251006160. (3) Hyperlink to the registration (must be publicly accessible): https://www.crd.york.ac.uk/PROSPERO/view/CRD420251006160.

## Use of artificial intelligence

Generative AI (ChatGPT, OpenAI, GPT-5, 2025 release) was used solely for language editing, grammar refinement, and formatting consistency. No patient-level or confidential data were provided as input. Only de-identified manuscript text was entered, and all outputs were reviewed and revised by the authors to ensure accuracy, clinical validity. AI was not involved in study design, data extraction, statistical analysis, or interpretation, and therefore did not influence the scientific findings of this meta-analysis. All stages of data synthesis and meta-analysis were conducted independently by the authors. The authors confirm the absence of conflicts of interest or financial ties to AI vendors and accept full responsibility for all content of the manuscript.

*Note*: A pilot systematic review and meta-analysis on the proportions of NF caused by emerging Gram-negative bacteria (studies published before 31 January 2019) served as the master’s thesis of the fifth author, Hsin Yang (National Taiwan University, 2019; advisor: Chi-Tai Fang).

## References

[1] Stevens DL Bryant AE Longo DL. Necrotizing soft-tissue infections. N Engl J Med 2017;377:2253–65. 29211672 10.1056/NEJMra1600673

[2] Hua C Urbina T Bosc R. Necrotising soft-tissue infections. Lancet Infect Dis 2023;23:e81–e94. 36252579 10.1016/S1473-3099(22)00583-7

[3] Wong CH Chang HC Pasupathy S Khin LW Tan JL Low CO. Necrotizing fasciitis: clinical presentation, microbiology, and determinants of mortality. J Bone Joint Surg Am 2003;85:1454–60. 12925624

[4] Allaw F Wehbe S Kanj SS. Necrotizing fasciitis: an update on epidemiology, diagnostic methods, and treatment. Curr Opin Infect Dis 2024;37:105–11. 38037890 10.1097/QCO.0000000000000988

[5] Kuehl R Tschudin-Sutter S Siegemund M. High mortality of non-fournier necrotizing fasciitis with enterobacteriales: time to rethink classification? Clin Infect Dis 2019;69:147–50. 30534983 10.1093/cid/ciy1011

[6] Stevens DL Bisno AL Chambers HF. Practice guidelines for the diagnosis and management of skin and soft tissue infections: 2014 update by the infectious diseases society of America. Clinl Infect Dis 2014;59:e10–e52. 10.1093/cid/ciu444 24973422

[7] Morgan MS. Diagnosis and management of necrotising fasciitis: a multiparametric approach. J Hosp Infect 2010;75:249–57. 20542593 10.1016/j.jhin.2010.01.028

[8] Tatara AM. Musculoskeletal Infection. In: Tatara AM, ed. The Infectious Diseases Consult Handbook: Common Questions and Answers. Cham: Springer International Publishing; 2023. 303–26.

[9] Cheng NC Cheng Y Tai HC. High mortality risk of type III monomicrobial gram-negative necrotizing fasciitis: the role of extraintestinal pathogenic Escherichia coli (ExPEC) and Klebsiella pneumoniae. Int J Infect Dis 2023;132:64–71. 37059297 10.1016/j.ijid.2023.04.390

[10] Yahav D Duskin-Bitan H Eliakim-Raz N. Monomicrobial necrotizing fasciitis in a single center: the emergence of Gram-negative bacteria as a common pathogen. Int J Infect Dis 2014;28:13–16. 25220388 10.1016/j.ijid.2014.05.024

[11] Park SY Yu SN Lee EJ. Monomicrobial gram-negative necrotizing fasciitis: an uncommon but fatal syndrome. Diagn Microbiol Infect Dis 2019;94:183–87. 30713116 10.1016/j.diagmicrobio.2018.12.013

[12] Kim HS Chang YJ Chung CH. Klebsiella pneumoniae necrotizing fasciitis on the upper lip in a patient with uncontrolled diabetes. Archi Craniofacial Surg 2020;21:127–31. 10.7181/acfs.2019.00696 PMC7206462 32380815

[13] Page MJ McKenzie JE Bossuyt PM. The PRISMA 2020 statement: an updated guideline for reporting systematic reviews. Int J Surg 2021;88:105906. 33789826 10.1016/j.ijsu.2021.105906

[14] Shea BJ Reeves BC Wells G. AMSTAR 2: a critical appraisal tool for systematic reviews that include randomised or non-randomised studies of healthcare interventions, or both. Bmj 2017;358:j4008. 28935701 10.1136/bmj.j4008 PMC5833365

[15] Riaz AA Ginimol M Rasha R. Transparency in the Reporting of Artificial Intelligence – the TITAN Guideline. Prem J Sci 2025;10:100082.

[16] Sterne JAC Savović J Page MJ. RoB 2: a revised tool for assessing risk of bias in randomised trials. Bmj 2019;366:l4898. 31462531 10.1136/bmj.l4898

[17] Wells GA Wells G Shea B. editors. The Newcastle-Ottawa Scale (NOS) for assessing the quality of nonrandomised studies in meta-analyses. 2014.

[18] Whiting PF Rutjes AWS Westwood ME. QUADAS-2: a revised tool for the quality assessment of diagnostic accuracy studies. Ann Intern Med 2011;155:529–36. 22007046 10.7326/0003-4819-155-8-201110180-00009

[19] Patsopoulos NA Evangelou E Ioannidis JP. Sensitivity of between-study heterogeneity in meta-analysis: proposed metrics and empirical evaluation. Int J Epidemiol 2008;37:1148–57. 18424475 10.1093/ije/dyn065 PMC6281381

[20] Furuya-Kanamori L Barendregt JJ Doi SAR. A new improved graphical and quantitative method for detecting bias in meta-analysis. Int J Evid Based Healthc 2018;16:195–203. 29621038 10.1097/XEB.0000000000000141

[21] Lee C-Y Kuo L-T Peng K-T Hsu W-H Huang T-W Chou Y-C. Prognostic factors and monomicrobial necrotizing fasciitis: gram-positive versus gram-negative pathogens. BMC Infect Dis 2011;11:5. 21208438 10.1186/1471-2334-11-5 PMC3022716

[22] Huang T-Y Peng K-T Hsiao C-T. Predictors for gram-negative monomicrobial necrotizing fasciitis in southern Taiwan. BMC Infect Dis 2020;20:60. 31959118 10.1186/s12879-020-4796-3 PMC6972015

[23] Sati H Carrara E Savoldi A. The WHO Bacterial Priority Pathogens List 2024: a prioritisation study to guide research, development, and public health strategies against antimicrobial resistance. Lancet Infect Dis 2025;25:1033–43. 10.1016/S1473-3099(25)00118-5 PMC12367593 40245910

[24] Kellum JA Ronco C. The role of endotoxin in septic shock. Crit Care 2023;27:400. 37858258 10.1186/s13054-023-04690-5 PMC10585761

[25] Qin S Xiao W Zhou C. Pseudomonas aeruginosa: pathogenesis, virulence factors, antibiotic resistance, interaction with host, technology advances and emerging therapeutics. Signal Transduct Target Ther 2022;7:199. 35752612 10.1038/s41392-022-01056-1 PMC9233671

[26] Javed A Balhuizen MD Pannekoek A. Effects of Escherichia coli LPS structure on antibacterial and anti-endotoxin activities of host defense peptides. Pharmaceuticals (Basel) 2023;16:1485. 10.3390/ph16101485 PMC10609994 37895956

[27] Patel PK Russo TA Karchmer AW. Hypervirulent Klebsiella pneumoniae. Open Forum Infect Dis 2014;1:ofu028. 25734101 10.1093/ofid/ofu028 PMC4324179

[28] Hakkarainen TW Kopari NM Pham TN Evans HL. Necrotizing soft tissue infections: review and current concepts in treatment, systems of care, and outcomes. Curr Probl Surg 2014;51:344–62. 25069713 10.1067/j.cpsurg.2014.06.001 PMC4199388

[29] Tsai YH Huang TY Kuo LT Chuang PY Hsiao CT Huang KC. Comparison of surgical outcomes and predictors in patients with monomicrobial necrotizing fasciitis and sepsis caused by vibrio vulnificus, aeromonas hydrophila, and aeromonas sobria. Surg Infect (Larchmt) 2022;23:288–97. 35180367 10.1089/sur.2021.337

[30] Meara JG Leather AJ Hagander L. Global Surgery 2030: evidence and solutions for achieving health, welfare, and economic development. Int J Obstet Anesth 2016;25:75–78. 26597405 10.1016/j.ijoa.2015.09.006

[31] Ronan D Holt H Utsi L. Localised increase in necrotising fasciitis associated with a shift to monomicrobial aetiology, South Yorkshire, England, 2023. J Infect 2025;90:106505. 40374090 10.1016/j.jinf.2025.106505

[32] Tsai YH Huang KC Liao WH Hsu RW Hsu WH. Liver cirrhosis as a risk factor for monomicrobial gram-negative necrotizing fasciitis. BMC Infect Dis 2019;19:1046–.31822287

[33] Baker-Austin C Trinanes JA Taylor NGH Hartnell R Siitonen A Martinez-Urtaza J. Emerging Vibrio risk at high latitudes in response to ocean warming. Nat Clim Change 2013;3:73–77.

[34] Huayllani MT Cheema AS McGuire MJ Janis JE. Practical review of the current management of Fournier’s Gangrene. Plast Reconstr Surg Glob Open 2022;10:e4191. 35295879 10.1097/GOX.0000000000004191 PMC8920302

[35] Kalil AC Metersky ML Klompas M. Management of adults with hospital-acquired and ventilator-associated pneumonia: 2016 clinical practice guidelines by the infectious diseases Society of America and the American Thoracic Society. Clin Infect Dis 2016;63:e61–e111. 27418577 10.1093/cid/ciw353 PMC4981759

[36] Howroyd F Chacko C MacDuff A. Ventilator-associated pneumonia: pathobiological heterogeneity and diagnostic challenges. Nat Commun 2024;15:6447. 39085269 10.1038/s41467-024-50805-z PMC11291905

[37] Wagenlehner FME Pilatz A Weidner W Naber KG. Urosepsis: overview of the diagnostic and treatment challenges. Microbiol Spectr 2015;3:UTI-0003-2012. 10.1128/microbiolspec.UTI-0003-2012 26542042

[38] Vincent JL Rello J Marshall J. International study of the prevalence and outcomes of infection in intensive care units. Jama 2009;302:2323–29. 19952319 10.1001/jama.2009.1754

[39] Laxminarayan R Matsoso P Pant S. Access to effective antimicrobials: a worldwide challenge. Lancet 2016;387:168–75. 26603918 10.1016/S0140-6736(15)00474-2

